# Determinants of poor adherence to antiretroviral treatment using a combined effect of age and education among human immunodeficiency virus infected young adults attending care at Letaba Hospital HIV Clinic, Limpopo Province, South Africa

**DOI:** 10.11604/pamj.2019.32.37.17722

**Published:** 2019-01-18

**Authors:** Kuhlula Mabunda, Emery Ladi Ngamasana, Joseph Omoniyi Babalola, Moleen Zunza, Peter Nyasulu

**Affiliations:** 1 Division of Epidemiology and Biostatistics, Department of Global Health, Faculty of Medicine and Health Sciences, Stellenbosch University, Cape Town, South Africa; 2 Carolina Population Centre, University of North Carolina at Chapel Hill, USA; 3 Division of Community Paediatrics, Faculty of Health Sciences, University of the Witwatersrand, Johannesburg, South Africa

**Keywords:** Adherence, antiretroviral treatment, HIV, Limpopo, South Africa

## Abstract

**Introduction:**

sustained viral suppression using antiretroviral treatment (ART) occurs with adherence to treatment of at least 95%. Non-adherence promotes the development of drug-resistance and treatment failure in individuals infected with Human Immunodeficiency Virus. In Limpopo Province, the adherence rate is approximately 61%, but the prevalence and the factors associated with adherence at Letaba hospital HIV clinic are not well established. Therefore, the aim of this study was to identify the factors associated with adherence among HIV-infected young adults, aged 18-35 years, attending the clinic.

**Methods:**

a cross-sectional survey was conducted in Letaba HIV clinic among young adults of 18-35 years old. Logistic regression analysis was performed to determine factors associated with ART adherence. We reported odds ratios with the corresponding 95% confidence intervals and p-values. A p-value < 0.1 was considered as statistically significant. ART adherence was defined as taking more than 95% of the prescribed treatment, 3 days prior to completion of the questionnaire.

**Results:**

a total of 281 participants were enrolled with 163 (58.0%) females and more than three quarter, 222 (79.0%) between the ages of 18 and 29 years. The overall ART adherence stood at (87.2%) (95% CI: 63.0%-89.0%) representing 245 participants. Non-adherers to treatment, 36 (12.8%): patients reported no reason (3.9%), forgetting (3.2%), feeling good (3.2%), fear and running out of treatment (2.5%) as some of the reasons for not taking treatment within the three days prior to data collection. The following factors: tertiary education (p = 0.07), age (30-35; p-value: 0.07), drug availability (p-value: 0.07), were only marginally significantly associated with ART adherence.

**Conclusion:**

the study found unsatisfactory ART adherence among our participants. Our study suggests that factors other than sociodemographic and clinical factors might better explain differences in adherence. This highlights the need for a more complex study that would look at the entire system in which these patients are navigating as well as their mental models.

## Introduction

Human Immunodeficiency Virus (HIV) that causes HIV infection and Acquired Immunodeficiency Syndrome (AIDS), remains one of the biggest global public health challenge in world history [1]. In 2016, approximately 1.8 million adults were newly infected with HIV, contributing to an estimated 36.7 million people living with HIV globally [2, 3]. Sub-Saharan Africa is the hardest hit, accounting for 70% of all people living with HIV globally [4]. South Africa has the highest number of people living with HIV in the world (7.06 million) and approximately 3.9 million adults have access to antiretroviral treatment (ART) of which approximately 260,893 are in Limpopo Province [5-7]. The roll-out of ART in Limpopo Province started in 2004 at the regional hospitals and is now freely available at all levels of health care facilities run by government and non-governmental organisations [7]. Access to ART alone is not enough to achieve the maximum health benefits. Patients must adhere to the treatment regimens at a rate of at least 95% [8]. The adherence rate to ART in Limpopo Province is approximately 61% [9], which is lower than the expected adherence rate of 95% as determined by the World Health Organization (WHO) [8]. Antiretroviral treatment suppresses the rate at which HIV multiplies within the body, making a person less susceptible to opportunistic infections as well as prevent the transmission of the virus to other individuals [8]. Failure to follow the prescribed ART regimen results in the development of drug-resistant strains of HIV and affects the immediate health outcome of the individual. This poses a threat of treatment failure and a financial burden on the health care system because other treatment options must be explored [8].

In 2013, the WHO released the treatment guidelines that stipulated that people who are HIV positive must start ART as early as possible, with a criterion of CD4 count less than or equal to 500 cells/mm3 compared to previous recommendation of CD4 count of 350 cells/mm3 or less [10]. By 2015, the treatment guidelines shifted to testing and treatment of anyone infected with HIV [11]. Starting ART early, increases the period an individual will be on treatment, and this may in addition to other factors impact on treatment adherence [12,13]. Previous studies indicated that patients on ART failed to reach optimal adherence due to reasons such as stigma, cultural beliefs, misinformation, lack of counselling, and travelling long distances to health facilities [14-16]. These determinants differ depending on social background and setting of individuals [16, 17]. HIV primarily affects those in their reproductive years; with 38% of the new infections reported in 2014 occurring among those under the age of 25 [2]. Recent studies also found that HIV infection is more prevalent among young adults between the ages of 18 and 30 years, a group which adheres less to ART due to socio-economic barriers [18]. The prevalence of adherence to ART and the factors associated with adherence among young adults at Letaba hospital HIV clinic are not well established and thus raises a public health concern because Letaba Hospital HIV clinic is within Mopani District Municipality which is the second district in Limpopo Province with the highest number of HIV/AIDS cases [9]. Therefore, the aim of this study was to determine the prevalence of adherence to ART and to identify the factors associated with ART adherence among HIV positive individuals attending the Letaba hospital HIV clinic. The findings of this study will be used to enhance current adherence programs to ART and establish new interventions targeting these determinants in order to increase patients' adherence to ART.

## Methods

**Study design:** the study employed a cross-sectional design to collect data on prevalence and associated factors influencing adherence to ART.

**Study population and sample size:** HIV-infected young adults between the ages 18 and 35 years on ART for three or more months were eligible for study participation. Pregnant women were eligible. Therefore, based on the current adherence level of 61% in Limpopo [9] allowing for a 5% margin of error with 95% confidence interval, a sample size of 281 patients was required. From the medical records used, the first participant was selected at random, followed by systematic random sampling. Every 3rd participant who met the inclusion criteria was eligible to participate in the study.

**Study measurements and procedures:** study participants were interviewed by trained research assistants using a structured questionnaire that was adapted from the Adult AIDS Clinical Trials Group (AACTG) [19]. Questions were formulated to gather the relevant information on the determinants of adherence (exposure variables) which include: sociocultural and demographic as well as patient and environmental factors. The outcome of interest was a binary variable defined as good or poor adherence. Patients were categorized as poor adherer if he/she missed a pill, or a pill per dose 3 days prior to the interview and good adherance if otherwise.

**Data analysis:** descriptive statistics including frequency and percentages were used to describe the characteristics of study participants. Logistic regression analysis was performed to determine factors associated with ART adherence. ART adherence was defined as taking more than 95% of the prescribed treatment 3 days prior to completion of the questionnaire. ART adherence was self-reported. We reported odds ratios with the corresponding 95% confidence intervals and p-values. A p-value <0.1 was considered as statistically significant. Variables that were statistically significant at 15% level of significance in the univariate analysis were included in the multivariate model. In allowing for such a high level of significance in the univariate analysis we wanted to account for the other missing contextual factors and as well as the limitations of the study. As argued by David Jean Biau *et al.* [20] it is important to consider a level of significance that accounts for the whole context (scientific relevance, economic considerations, aims and limitations of the studies). Given that HIV itself is a highly sensitive topic, we decided not to be so conservative to account for the interplay of social desirability in the responses provided by participants, which is one of the biggest limitations of this study.

**Ethical approval:** ethical clearance was obtained from University of Stellenbosch (certificate number S16/07/214) and the National Health Research Database after approval by the Department of Health, Limpopo Province. Written informed consent was obtained from each of the participants prior to data collection.

## Results

A total of 281 patients participated in the study of which 163 (58.0%) were females and 118 (42.0%) were males on ART for a minimum of 3 months prior to the study. Two hundred and twenty-two (79.0%) participants were between the ages 18 and 29 years. One hundred and sixty-eight (59.8%) were single, this included the divorced and the widowed and 113 (40.2%) were married or cohabitating. Forty-three (15.3%) never attended school. Most of the participants had primary and secondary education 226 (79.9%) and 12 (4.3%) had tertiary education. Most (76.5%) of the participants were unemployed. Majority of the participants (59.4%) did not receive any monthly income while 33.5% and 7.1% earned monthly income of less than R2,999 and between R3,000 and R10,000 respectively. Two hundred and fifty-three (90.0%) live less than 10km from the clinic, therefore they walk or use mini bus taxis to get to the clinic. About 21.4% and 35.9% of the participants were smoking and taking alcohol, respectively (Table 1).

**Table 1:** sociocultural and demographic characteristic of the study participants

Characteristics	Frequency	%
**Age**		
18 - 23	110	39.2
24 - 29	112	39.8
30 - 35	59	21.0
**Gender**		
Male	118	42.0
Female	163	58.0
Marital Status		
Single / Divorce / Widow	168	59.8
In union	113	40.2
**Religion**		
Christians	141	50.2
Others	140	49.8
**Language**		
Xitsonga	160	56.9
Sepedi	111	39.5
Others	10	3.6
**Education**		
None	43	15.3
Primary	81	28.8
Secondary	145	51.6
Tertiary	12	4.3
**Employment Status**		
Unemployed	215	76.5
Employed	66	23.5
**Income Categories**		
No Income	167	59.4
R100 - R2999	94	33.5
R3000 -R10000	20	7.1
**Distance from Healthcare Facility**		
< 5Km	93	33.1
5-10Km	160	56.9
11-20Km	28	10.0
**Alcohol Status**		
Non drinker	180	64.1
Drinker	101	35.9
**Smoking Status**		
Non Smoker	221	78.7
Smoker	60	21.3

In the assessment of patient-related factors and environmental factors, 245 (87.2%) (95% CI: 63.0%-89.0%) participants adhered to treatment. There were 190 (67.2%) participants on ART for more than two years and 215 (75.8%) had disclosed their HIV status to their spouse, family, friends and colleagues. No side effect was experienced while on treatment by 250 (89.0%) of the participants. Illness, a month prior to the study, was low with 36 (12.8%) reporting to have been ill. The participants did not generally use any form of reminder to take treatment, only 84 (29.9%) utilised an alarm, a certain program on television or friend to remind them to take their treatment. Those that did not adhere to treatment 36 (12.8%) reported forgetting (3.2%), feeling good (3.2%), fear and running out of treatment (2.5%) as some of the reasons for not taking treatment in the past three days prior to data collection. The patient and health care provider relationships were good with 262 (93.2%) of the patients, reporting that they trust and have positive attitudes to their health care providers and believe that their HIV statuses will be kept confidential (Table 2). In the univariate analysis of factors associated with adherence, education (p=0.104), use of reminder to take treatment (p=0.142), alcohol consumption (p=0.131), disease severity (p=0.084) and drug availability (p=0.066) were significantly associated with adherence to ART at 0.1% level of significance (Table 3).

**Table 2:** patients related and environmental factors

Patients related factors	Frequency	%
**Method of reminder**		
None	197	70.1
Alarm	35	12.5
TV	38	13.5
Friend	11	3.9
**Disclosure**		
No	68	24.2
Yes	213	75.8
**Feeling status**		
Bad	64	22.8
Fair	41	14.6
Good	176	62.6
**Duration under ART**		
< 1 year	63	22.4
1 - 2 years	28	10.0
2 years +	190	67.6
**Failed adherence**		
No	245	87.2
Yes	36	12.8
**Reported side effects**		
No	250	89.0
Yes	31	11.0
**Illness severity**		
None	250	89.0
Mild	23	8.2
Severe	8	2.8
**ART change**		
No	163	58.0
Yes	118	42.0
**Illness**		
No	245	87.2
Yes	36	12.8
**Attitude**		
Negative	20	7.1
Positive	261	92.9
**Waiting time (queuing)**		
< 1 hour	163	58.0
1 - 2 hours	112	39.9
2 - 3 hours	6	2.1
**Use of reminder**		
No	197	70.1
Yes	84	29.9

**Table 3:** univariate analysis of factors associated with poor adherence to antiretroviral treatment

Characteristics	Adherence Status		p-value
	poor	good	
**Age**			
18 – 23	16 (14.6)	94 (85.4)	0.296
24 – 29	16 (14.3)	96 (85.7)	
30 – 35	4 ( 6.8)	55 (93.2)	
**Gender**			
Male	18 (15.2)	100 (84.8)	0.297
Female	18 (11.0)	145 (89.0)	
**Marital status**			
Single / Divorce / Widow	21 (12.5)	147 (87.5)	0.849
In union	15 (13.3)	98 (86.7)	
**Education**			
None	3 ( 7.0)	40 (93.0)	0.104
Primary	9 (11.1)	72 (88.9)	
Secondary	20 (13.8)	125 (86.2)	
Tertiary	4 (33.3)	8 ( 66.7)	
**Distance from healthcare facility**			
< 5Km	12 (12.9)	81 (87.1)	0.939
5-10Km	21 (13.1)	139 (86.9)	
11-20Km	3 (10.7)	25 (89.3)	
**Disclosure status**			
No	10 (14.7)	58 (85.3)	0.591
Yes	26 (12.2)	187 (87.2)	
**Duration of treatment**			
< 1 year	10 (15.9)	53 (84.1)	0.261
1 – 2 years	1 (3.6)	27 (96.4)	
2 years +	25 (13.2)	165 (86.8)	
**Side effects**			
No	30 (12.0)	220 (88.0)	0.248
Yes	6 (19.4)	25 (80.6)	
**Disease severity**			
None	30 (12.0)	220 (88.0)	0.084
Mild	6 (26.1)	17 (73.9)	
Severe	0 (0.0)	8 (100.0)	
**ART change**			
No	21 (12.9)	142 (87.1)	0.966
Yes	15 (12.7)	103 (87.3)	
**Drug unavailability**			
Yes	34 (12.3)	242 (87.7)	0.066
No	2 (40.0)	3 (60.0)	

A logistic regression model was performed to document the likelihood of adherence, using a set of covariates (predictors significant at 15% level of significance in the bivariate analysis (Table 4). By allowing for such a high level of statistical significance, we accounted for the whole context, acknowledging that we did not control for many other contextual factors that may affect lack of adherence. Besides, given the relatively highly sensitive nature of the topic, we expected participant's responses to suffer from social desirability biases. In the bivariate logistic regression model, having a tertiary education (OR: 6.67; 95% C.I: 1.24−35.71) was statistically associated with increased likelihood of failure to adhere to treatment. Although factors such as alcohol consumption and use of reminder methods were not statistically significant at 10%, we kept them in the covariate-adjusted model, since they are relevant factors that have been widely reported as factors contributing to non-adherence. The other factors including waiting time, attitudes, ART change were insignificant at 10% level of significance, thus excluded from the multivariate logistic regression model.

**Table 4:** likelihood for adherence using selected socio-demographic and clinical factors (odds ratio and 95% Confidence Intervals)

Characteristics	Unadjusted logistic model	Adjusted logistic model
	OR (95% C.I)	OR (95% C.I)
**Age Category**		
<25	Ref	Ref
25-29	0.90 (0.42-1.93)	0.73 (0.30-1.76)
30-35	0.41 (0.13-1.27)	0.28 (0.07-1.17)
**Education**		
None	Ref	Ref
Primary	1.67 (0.43-6.51)	0.74 (0.15-3.71)
Secondary	2.13 (0.60-7.56)	1.15 (0.27-4.85)
Tertiary	6.67 (1.24-35.71)	5.85 (1.02-34.47)
**Reminders**		
No	Ref	Ref
Yes	0.53 (0.22-1.25)	0.53 (0.22-1.31)
**Severity of sickness**		
No	Ref	Ref
Yes	1.11 (0.40-3.08)	1.29 (0.44-3.81
**Alcohol Consumption**		
No	Ref	Ref
Yes	1.71 (0.85-3.47)	1.70 (0.80-3.59)
**Use of Reminder**		
No	Ref	Ref
Yes	0.53 (0.22-1.25)	0.53 (0.22-1.31)
**Drug Unavailability**		
No	Ref	Ref
Yes	4.75 (0.77-29.43)	3.56 (0.50-25.49)
**Distance to health facility**		
< 5Km	Ref	Ref
1-10Km	1.02 (0.48-2.18)	1.10 (0.49-2.46)
11-20Km	0.81 (0.21-3.10)	1.02 (0.24-4.35

**Figure 1:**
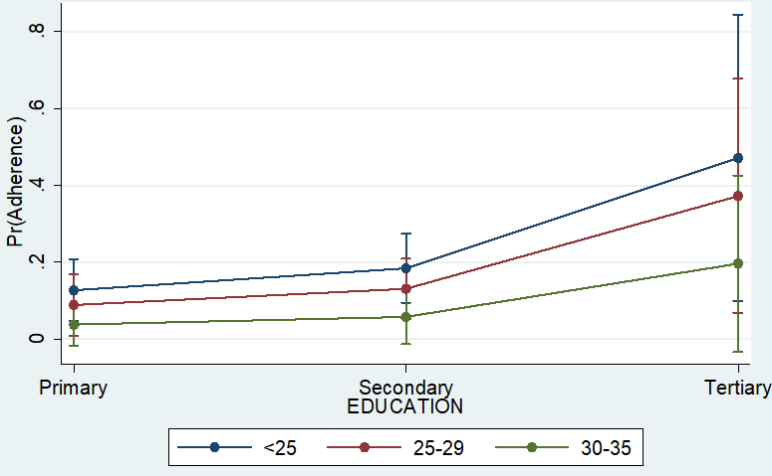
adherence prediction using a combined effect of age and education

**Figure 2:**
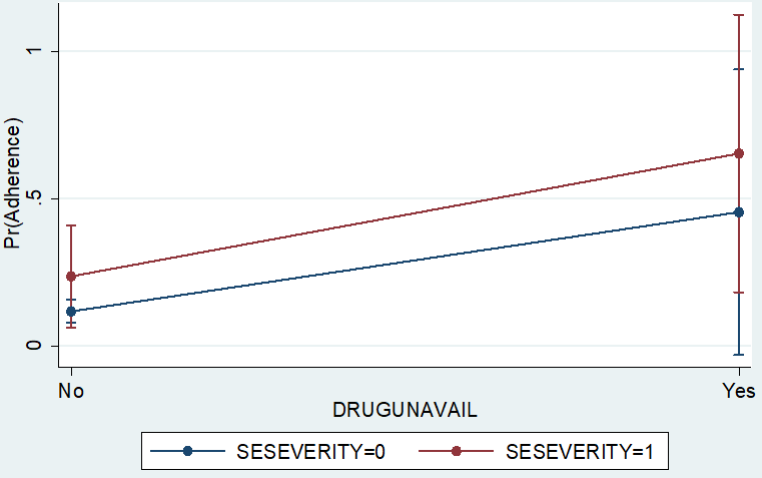
adherence prediction using a combined effect of disease severity and drug availability

Our study found that poor adherence decreased with age, while on the other hand, it increased with education (Figure 1). However, the overlap between the 95% confidence interval band widths suggests that such a difference is not consistently significant and may vanish in a larger sample. We found that younger and less educated patients were less likely to be poor adherers to treatment compared to older educated patients. In addition, we found that older patients (30−35 years old) were less likely to fail to adhere to treatment (OR: 0.29; 0.07−1.17) compared to patients less than 25 years of age. Although, drug availability showed huge differences (OR: 4.61; 95% C.I.: 0.68−31.01) in poor adherence among the study participants, however, this difference was not statistically significant, because poor adherence varied widely among those with medication as well (Figure 2). We also found that patients lacking drugs with moderate or advanced stage of the disease were less likely to be poor adherers, compared to similar patients who had access to the drugs. However, this difference was not significant because among those with drugs at early stage of the diseases, the likelihood of poor adherence varied widely, with some probabilities equaling that of patients with no drugs (Figure 2).

**Figure 3:**
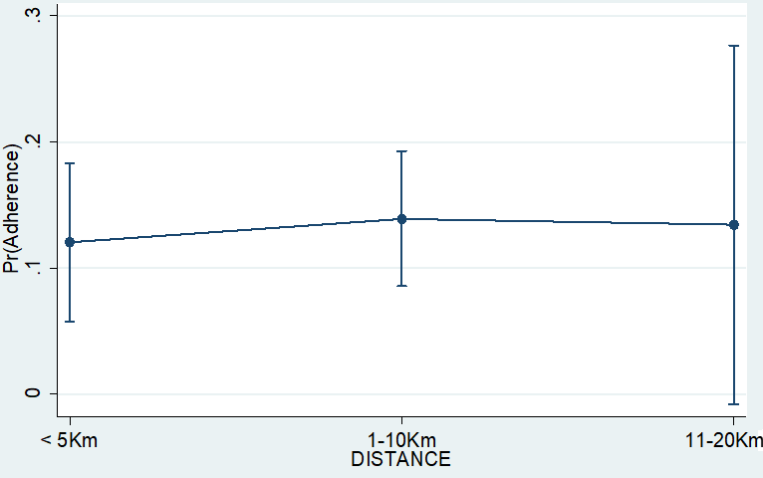
adherence prediction using the effect of distance to health facility

Finally, we found also that the likelihood of poor adherence varied based on the distance that the patients had to travel to the health facility (Figure 3). Generally, those close to the health facility were less likely to adhere (margins: 0.12). However, such a difference was not statistically significant because we also found that adherence among those living far from the health facility varied widely with some being to the health facility displayed some variations.

## Discussion

The prevalence of adherence to ART in this study was 87% (95% CI: 63.0%-89.0%), indicating that 13 in every 100 participants were non-adherent. This adherence level, is higher than the Limpopo provincial rate of 61.0% [9]. This implies that the 13% of the patients with poor adherance status were still unsatisfactory when compared to the adherence benchmark of 95%, as determined by the WHO, which is required for sustained viral load suppression [8]. High adherence to ART, which is taking 95% of the prescribed medication, suppresses the viral load to undetectable levels thereby reducing the disease progression and the risk of transmission of the virus to others [20]. The proportion of non-adherence seen in this study, can result in massive drug resistance and reversal of the progress achieved in the control of the HIV epidemic [21, 22]. This will pose an additional financial burden on the already limited health resources.

There was high drug availability, though 5 (1.8%) participants, 2 from the non-adherent group and 3 from the adherent group, reported being turned back without treatment due to drug unavailability. The following factors were associated with adherence in this study: improved health status, stigma, running out of medical supplies and forgetting to take treatment. Improved health status affected the participants' adherence to treatment in that they stopped taking their medication [23]. Our results were similar to those of Charurat *et al.* 2010, in a study done in Nigeria that reported that improved health could result in non-adherence if people believed they had been cured [24]. About two third (62.63%) of patients reported having good feeling, while 22% reported feeling bad about their status. While we acknowledge that stigma has been widely reported as a factor for non-adherence, in our study this did not come out clearly. As found in other studies, stigma is likely to provoke discrimination, rejection and isolation of people living with HIV [25]. It can also contribute to low rate of disclosure and thus poor adherence [25, 26]. In our study, we found a high percentage of disclosure (75.8%). This suggests that the study participants are generally accepting their condition and fears of stigma do not impact their retention and or adherence to treatment. Other research suggests that fear of stigma and discrimination influence some patients avoid going to hospital to replenish their drug supply and thus interrupt their treatment [15].

However, we believe that stigma, in this study, created emotional distress that resulted in some patients drinking and smoking heavily, as a way of providing some self-comfort. For instance, adherence to treatment was relatively higher among non-drinkers (89.4%), compared to drinkers (83.2%). In other words, alcohol consumption was associated with lower levels of adherence to ART among patients. However, in the final covariate adjusted model, we found very little evidence that poor adherence was ever affected by alcohol drinking status. A better alternative was to consider including alcohol intake frequency in the model, but this was not done simply because of low records at the lower stratification (no alcohol intake, daily, weekly). Nevertheless, the study showed some association between alcohol intake status and poor adherence to treatment. Better data could spark such evidence. Kekwaletswe *et al.* 2014 had similar findings, which supports the hypothesis that alcohol consumption is associated with low levels of ART adherence [27]. Another disadvantage of this habit is that patients may forget to take their medication because of alcohol misuse. Men may also indulge in alcohol to deny the reality of being HIV-positive [28]. In this population those that failed to adhere to treatment were higher in the more educated groups, those with secondary (13.79%) and tertiary (33.3%) education compared to those without education (7.0%). This is different from the observation in most other studies that reported non-adherence among individuals with lower levels of education [29]. However, others had findings similar to ours [30]. Being educated does not mean that an individual is equipped with relevant knowledge relating to ART but this knowledge can be acquired through patient and health care provider interactions [31]. This was supported by a study conducted in Malawi that reported that appropriate health education was a useful approach to increasing patient's adherence because patients were taught about proper ART guidelines before they were commenced on medication [32].

From our study, we also found a statistically significant association between illness in the past month and poor adherence to ART. Among the patients who failed to adhere to treatment, those that experienced mild and severe illness were found at a higher likelihood of suboptimal adherence than those who were healthy. According to a study conducted by Markos *et al.* 2008, having symptoms in the past one month was associated with non-adherence, which is in line with our findings [33]. Another factor that was found to have a significant association with poor adherence to ART was the use of reminders to take medication. Most research reports that patients who use reminders to take medication were more likely to have better adherence than those who did not use any reminders [16, 34, 35]. Patients who use memory aids were three times more likely to adhere to treatment than those who did not use any memory aids [34]. Among the adherent group in our study, those that use reminders to take treatment (91.7%) were more adherent than those that did not use any reminders (85.3%). This suggests that patients should be encouraged to use reminders regularly to ensure that they take medications as prescribed [36]. However, among the non-adherent group, those that used reminders to take treatment (8.3%), adhered less compared to those that did not use any reminder (14.7%). This suggests that other factors are involved, other than having reminders among the non-adherents. This study was based on self-reported data on missed doses. Self-reports are subject to social desirability and recall bias [37] as evidenced by previous literature showing that self-reports tend to over-estimate adherence [38].

## Conclusion

The prevalence of adherence to ART in this study (87.0%) was unsatisfactory, though higher than the Limpopo provincial rate (61.0%), it was less than the 95% recommended by WHO. The study also revealed that the following factors were negatively associated with ART adherence: improved health status, stigma, running out of medication, forgetting to take treatment, illness and lack of facilities for treatment reminders. These factors need to be addressed to alleviate concerns of drug resistance and the resultant heavy financial burden on the health systems from the need to control resistant strains of HIV. Another negative implication of poor adherence is total treatment failure with increased morbidity and mortality. Efforts by health care providers must be improved in counselling at initiation and during follow-up hospital visits. The health care providers should regularly counsel and educate patients before initiation and during the course of ART. The counselling sessions should emphasize on the nature of the HIV disease, characteristics of HIV medication, benefits of ART, the importance of adherence, the goal of the treatment, and the risks of non-adherence to ART. However, this requires a concerted effort among the multidisciplinary team, including adherence counsellors.

### What is known about this topic

- There is increasing access to ART program among HIV positive individuals and with it comes the challenge of treatment adherence;
- Adherence to ART among HIV positive individuals is a key success factor as it enhances viral suppression, decreases the risk of HIV transmission, reduces the risk of ART drug resistance and improves the quality of life.

### What this study adds

- Adherence to ART in this study was unsatisfactory, though higher than the previously reported rate for the Limpopo provincial rate, it was lower than the rate recommended by the WHO;
- Factors that were negatively associated with ART adherence among individuals attending care at Letaba hospital included ‘improved health status, HIV related stigma, running out of medication, lack of facilities for treatment reminders’ among individuals in HIV care;
- The need to reinforce information, education and communication practices in this setting in order to expand awareness and knowledge regarding negative effect of poor adherence to ART at the time of treatment initiation.

## References

[1] World Health Organization (2015). Global Health Observatory (GHO) data 2015.

[2] UNAIDS, Joint United Nations Programme on HIV and AIDS (2017). Factsheet July 2017.

[3] UNAIDS, Joint United Nations Programme on HIV and AIDS (2017). UNAIDS DATA 2017.

[4] Kharsany ABM, Karim QA (2016). HIV infection and AIDS in Sub-saharan Africa: current status, challenges and opportunities. Open AIDS J.

[5] Africa SS, Statistics South Africa (2017). Statistical release p0302 mid-year population estimates 2017.

[6] Avert (2018). HIV and AIDS in South Africa.

[7] South African National AIDS Council (SANAC), Limpopo Provincial AIDS Council (LPAC) Annual progress report 2015/16. Provincial Strategic Plan (PSP) 2012-2016.

[8] World Health Organization (2010). Antiretroviral therapy for HIV infection in adults and adolescents: recommendations for a public health approach.

[9] Department of Health Limpopo (2015). Operational plan on comprehensive HIV and AIDS Care.

[10] World Health Organization (2013). Consolidated guidelines on the use of antiretroviral drugs for treating and preventing HIV infection: recommendations for a public health approach.

[11] World Health Organization (2016). Consolidated guidelines on the use of antiretroviral drugs for treating and preventing HIV infection: recommendations for a public health approach.

[12] Chaiyachati KH, Ogbuoji O, Price M, Suthar AB, Negussie EK, Bärnighausen T (2014). Interventions to improve adherence to antiretroviral therapy: a rapid systematic review. AIDS.

[13] Thompson MA, Mugavero MJ, Amico K (2012). Guidelines for improving entry into and retention in care and antiretroviral adherence for persons with hiv: Evidence-based recommendations from an international association of physicians in aids care panel. Ann Intern Med.

[14] Xu L, Munir K, Kanabkaew C, Le Coeur S (2017). Factors influencing antiretroviral treatment suboptimal adherence among perinatally HIV-infected adolescents in Thailand. PloS One.

[15] Ndiaye M, Nyasulu P, Nguyen H (2013). Risk factors for suboptimal antiretroviral therapy adherence in HIV-infected adolescents in Gaborone, Botswana: a pilot cross-sectional study. Patient Prefer Adherence.

[16] Heestermans T, Browne JL, Aitken SC, Vervoort SC, Klipstein-Grobusch K (2016). Determinants of adherence to antiretroviral therapy among HIV-positive adults in sub-Saharan Africa: a systematic review. BMJ Glob Health.

[17] Johnson MO, Chesney MA, Goldstein RB (2006). Positive provider interactions, adherence self-efficacy, and adherence to antiretroviral medications among HIV infected adults: a mediation model. AIDS Patient Care STDs.

[18] Dewing S, Mathews C, Fatti G, Grimwood A, Boulle A (2014). Antiretroviral adherence interventions in Southern Africa: implications for using HIV treatments for prevention. Curr HIV/AIDS Rep.

[19] Chesney MA, Ickovics J, Chambers D (2000). Self-reported adherence to antiretroviral medications among participants in HIV clinical trials: the AACTG adherence instruments. AIDS Care.

[20] Meintjes G, Conradie J, Cox V (2014). Adult antiretroviral therapy guidelines 2014. South Afr J HIV Med.

[21] Sabaté E (2003). Adherence to long-term therapies: evidence for action.

[22] Sethi AK, Celentano DD, Gange SJ, Moore RD, Gallant JE (2003). Association between adherence to antiretroviral therapy and human immunodeficiency virus drug resistance. Clin Infect Dis.

[23] Mthembu TG, Van Wyk B (2014). Patients' knowledge and beliefs about antiretroviral treatment and factors associated with adherence in Mpumalanga Province, South Africa. Health SA Gesondheid (Online).

[24] Charurat M, Oyegunle M, Benjamin R (2010). Patient retention and adherence to antiretrovirals in a large antiretroviral therapy program in Nigeria: a longitudinal analysis for risk factors. PloS One.

[25] Reda AA, Biadgilign S (2012). Determinants of adherence to antiretroviral therapy among HIV-infected patients in Africa. AIDS Res Treat.

[26] Bhat V, Ramburuth M, Singh M (2010). Factors associated with poor adherence to anti-retroviral therapy in patients attending a rural health centre in South Africa. Eur J Clin Microbiol Infect Dis.

[27] Morojele NK, Kekwaletswe CT, Nkosi S (2014). Associations between alcohol use, other psychosocial factors, structural factors and antiretroviral therapy (ART) adherence among South African ART recipients. AIDS and Behav.

[28] Hormes JM, Gerhardstein KR, Griffin PT (2011). Brief screening for alcohol use disorders in HIV primary care. HIV Clin.

[29] Tapp C, Milloy M, Kerr T (2011). Female gender predicts lower access and adherence to antiretroviral therapy in a setting of free healthcare. BMC Infect Dis.

[30] Boima V, Ademola AD, Odusola AO (2015). Factors associated with medication nonadherence among hypertensives in Ghana and Nigeria. Int J Hypertens.

[31] Venkatesh KK, Srikrishnan A, Mayer KH (2010). Predictors of nonadherence to highly active antiretroviral therapy among HIV-infected South Indians in clinical care: implications for developing adherence interventions in resource-limited settings. AIDS Patient Care STDS.

[32] McKinney O, Modeste NN, Lee JW, Gleason PC, Maynard-Tucker G (2014). Determinants of antiretroviral therapy adherence among women in southern Malawi: healthcare providers' perspectives. AIDS Res Treat.

[33] Markos E, Worku A, Davey G (2008). Adherence to ART in PLWHA and Yirgalem Hospital, South Ethiopia. Ethiop J Health Dev.

[34] Langebeek N, Gisolf EH, Reiss P (2014). Predictors and correlates of adherence to combination antiretroviral therapy (ART) for chronic HIV infection: a meta-analysis. BMC Med.

[35] Hornschuh S, Dietrich JJ, Tshabalala C, Laher F (2017). Antiretroviral treatment adherence: knowledge and experiences among adolescents and young adults in Soweto, South Africa. AIDS Res Treat.

[36] Mbuagbaw L, Thabane L, Ongolo-Zogo P (2012). Trends and determining factors associated with adherence to antiretroviral therapy (ART) in Cameroon: a systematic review and analysis of the CAMPS trial. AIDS Res Ther.

[37] Simoni JM, Kurth AE, Pearson CR, Pantalone DW, Merrill JO, Frick PA (2006). Self-report measures of antiretroviral therapy adherence: a review with recommendations for HIV research and clinical management. AIDS and Behav.

[38] Stirratt MJ, Dunbar-Jacob J, Crane HM (2015). Self-report measures of medication adherence behavior: recommendations on optimal use. Transl Behav Med.

